# Neuronal and non-neuronal scaling across brain regions within an intercross of domestic and wild chickens

**DOI:** 10.3389/fnana.2022.1048261

**Published:** 2022-11-25

**Authors:** Felipe Cunha, Diego Stingo-Hirmas, Rita France Cardoso, Dominic Wright, Rie Henriksen

**Affiliations:** Department of Physics, Chemistry and Biology, Linköping University, Linköping, Sweden

**Keywords:** brain allometry, brain size, neuronal density, intraspecific variation, within-population

## Abstract

The allometric scaling of the brain size and neuron number across species has been extensively studied in recent years. With the exception of primates, parrots, and songbirds, larger brains have more neurons but relatively lower neuronal densities than smaller brains. Conversely, when considering within-population variability, it has been shown that mice with larger brains do not necessarily have more neurons but rather more neurons in the brain reflect higher neuronal density. To what extent this intraspecific allometric scaling pattern of the brain applies to individuals from other species remains to be explored. Here, we investigate the allometric relationships among the sizes of the body, brain, telencephalon, cerebellum, and optic tectum, and the numbers of neurons and non-neuronal cells of the telencephalon, cerebellum, and optic tectum across 66 individuals originated from an intercross between wild and domestic chickens. Our intercross of chickens generates a population with high variation in brain size, making it an excellent model to determine the allometric scaling of the brain within population. Our results show that larger chickens have larger brains with moderately more neurons and non-neuronal cells. Yet, absolute number of neurons and non-neuronal cells correlated strongly and positively with the density of neurons and non-neuronal cells, respectively. As previously shown in mice, this scaling pattern is in stark contrast with what has been found across different species. Our findings suggest that neuronal scaling rules across species are not a simple extension of the neuronal scaling rules that apply within a species, with important implications for the evolutionary developmental origins of brain diversity.

## Introduction

The variation in brain size has been of long-standing interest to biologists due to the cognitive and behavioral phenotypes that such variation is thought to underlie. For example, numerous studies have attempted to determine the explanatory factors underlying brain size variation among species (Bennett and Harvey, 1985; Lefebvre et al., 2004; Van Schaik et al., 2012; Herculano-Houzel et al., 2014; Olkowicz et al., 2016; Sayol et al., 2016). Although absolute brain size varies more than 100,000-fold in vertebrates (Count, 1947; Herculano-Houzel, 2011a), for many years it was implied that the relationships among brain mass, body size, and number of neurons were universal (Haug, 1987; Finlay and Darlington, 1995; Van Dongen, 1998; Barton and Harvey, 2000). That is, increases in brain size were thought to reflect proportional changes in the numbers of neurons through a similar fashion across all species. In recent decades, however, data on many different species have revealed that the allometric relationship between brain size and number of neurons can vary from one taxon to another (Herculano-Houzel et al., 2014; Olkowicz et al., 2016; Dos Santos et al., 2017; Kverková et al., 2022). Moreover, when comparing species (except for primates, parrots, and songbirds), numbers of neurons increase more slowly than brain size, which is accompanied by a decrease in neuronal density (Herculano-Houzel et al., 2014; Olkowicz et al., 2016). Therefore, because different species with similar brain sizes can have different numbers of neurons (Herculano-Houzel et al., 2014; Olkowicz et al., 2016; Nìmec and Osten, 2020), it has been suggested that neuron numbers or neuronal density might be a better indicator of the brain processing capacity rather than just looking at size (Herculano-Houzel, 2017; Kverková et al., 2022).

Although there is a wealth of information about how brain size and neuronal composition vary between species (Herculano-Houzel et al., 2006, 2014; Neves et al., 2014; Dos Santos et al., 2017; Herculano-Houzel et al., 2020; Cunha et al., 2021; Kverková et al., 2022; Sol et al., 2022), far less is known about how these traits vary within populations (Herculano-Houzel et al., 2015a; Marhounová et al., 2019; Kverková et al., 2020). This is surprising, given that intra-species variation is likely the initial driver for inter-species variation during speciation (Grant, 1981; Danley and Kocher, 2001). When intra-species variation has been considered, opposing findings on the allometric scaling of the brain within-population have been reported (Herculano-Houzel et al., 2015a; Marhounová et al., 2019; Kverková et al., 2020). For example, larger individuals of geckoes (*Paroedura picta*) have significantly larger brains with more neurons (Kverková et al., 2020), and guppies (*Poecilia reticulata*) artificially selected for larger brains have proportionally more neurons in the brain than individuals with relatively smaller brains (Marhounová et al., 2019). Conversely, in mice (*Mus musculus*), brain size is not a good proxy for the numbers of neurons (Herculano-Houzel et al., 2015a).

To investigate the relationship between brain size and composition, neuronal number and neuronal density within a population, the degree of variation present in the population used is vital to be able to disentangle the relative effects of the traits on one another. Despite this, most studies to date analyze laboratory populations with relatively low variability in traits between individuals (Tramontin et al., 1998; Ward et al., 2001; Herculano-Houzel et al., 2015a; Marhounová et al., 2019), while only a few include different populations (Kverková et al., 2020). Unsurprisingly, the variation in brain anatomy is significantly larger across populations than within populations (Kverková et al., 2020; Reyes et al., 2022). In domesticated chickens, selection has led to extreme phenotypic changes in a wide variety of traits, but one of the core changes has been to brain size and composition (Wright, 2015; Henriksen et al., 2016; Wright et al., 2020; Racicot et al., 2021). Historically, considerable attention has been given to the size of the brain relative to body size in domesticated populations (Kruska, 1970, 1996, 2005; Ebinger and Röhrs, 1995). For instance, most domestic strains, including chickens, have decreased relative brain size when compared with their wild ancestors, a finding that has been interpreted as a reduction in overall brain size due to domestication (Kruska, 1970, 2005; Plogmann and Kruska, 1990; Ebinger and Röhrs, 1995). However, when comparing domestic chickens to their wild ancestor (red junglefowl), the domestication process has caused not only major increases in body size (85%) but also a 15% increase in the absolute size of the brain with proportional changes in all major brain regions (Henriksen et al., 2016). Moreover, the increase in absolute brain size (chiefly the cerebellum and telencephalon) in domestic chickens reflects changes in the microanatomy of the brain (i.e., cerebellum), such as number and size of neurons (Racicot et al., 2021). This means that, in the case of the domestication, the use of body size as a normalizer for determining how brain size changes granted the misunderstanding that brain size shrinks with domestication. Recently, by generating an advanced intercross population via intercrossing wild and domestic chickens and using it to map the genetic loci underpinning body size and brain size, these two traits were found to have an entirely separate genetic architecture, indicating that separate genetic loci regulate the population differences affecting the large inter-population variation (Henriksen et al., 2016). If the two populations had simply been compared with one another, these traits would appear to be potentially genetically pleiotropic, with the same genes influencing both traits. However, when an intercross population is generated, it is then possible to disentangle these effects as individuals become mosaics of wild and domestic genotypes, allowing us to ascertain if larger individuals always have larger brain sizes and to independently map these traits to assess if the same genotypes affect both brain size and body size.

In this study, by examining 66 individuals from an advanced intercross population between domestic (white leghorn) and wild (red junglefowl) chickens, we determine whether larger individuals have larger brains with more neurons and lower neuronal density. As mentioned above, domestic chickens have larger body sizes and brain sizes than their wild counterparts (Henriksen et al., 2016). Thus, an intercross between these two populations generates individuals with high variability within one population, allowing the relationships between brain size, body size, neuronal number and density to be disentangled and investigated with a far greater degree of precision than is possible in a standard population with relatively little variation present. For all individuals examined, we measured the weights of the body, brain, telencephalon, cerebellum, optic tectum, and brain remainder (thalamus, remaining midbrain, and hindbrain). For the telencephalon, cerebellum, and optic tectum, we quantified the numbers of neurons and non-neuronal cells. With this dataset, we were able to determine (1) whether the numbers of neurons and non-neuronal cells vary relative to brain size within a highly variable population of chickens and (2) if there are significant differences in the allometric relationships performed from one brain region to another. Based on comparative analyses of the brain anatomy among species and populations, we hypothesized that larger chickens would have larger brains with more neurons but relatively lower neuronal densities.

## Materials and methods

### Specimens

We obtained measurements from 66 individual chickens raised in two different batches and originated from an advanced intercross (F18) between one male of red junglefowl and three females of white leghorns (Stingo-Hirmas et al., 2022). Each intercross generation was maintained with ∼100 individuals per generation, and were specifically bred to maintain and maximize genetic variation. Both red junglefowl and white leghorn belong to the species *Gallus gallus*, with the difference that white leghorns (*G. gallus domesticus*) have been selected to lay eggs with maximum efficiency (Kerje et al., 2003). Of the 66 individuals, 41 were females and 25 were males (Supplementary Table 1). All individuals were raised following the same conditions as described in Stingo-Hirmas et al. (2022). At 229 days of age, the adult individuals had their body weights measured, followed by euthanasia *via* cervical neck dislocation and decapitation. The study was approved by the local Ethical Committee of the Swedish National Board for Laboratory Animals.

### Brain measurements

After culling, the brains of all individuals were immediately extracted and the following regions dissected: telencephalon, cerebellum, optic tectum, and brain remainder (thalamus, remaining midbrain, and hindbrain; Supplementary Figure 1). The brain regions were weighed immediately after dissection (Supplementary Table 1), and the right hemispheres of the telencephalon, optic tectum, and cerebellum (cut down in the vermis) were immersion fixed in 4% paraformaldehyde in 0.1 M phosphate buffer (*sensu* Stingo-Hirmas et al., 2022). The left hemisphere was flash-frozen in liquid nitrogen and stored in −80°C for posterior gene expression analyses. Given that the hypothalamus, in both hemispheres, had to be preserved for a parallel study, we were not able to determine the numbers of cells in the brain remainder. Hereafter, the masses of the body and brain are also referred to as sizes. For the telencephalon, cerebellum, and optic tectum, the numbers of neurons and non-neuronal cells were determined by following the isotropic fractionator technique (Herculano-Houzel and Lent, 2005; Supplementary Table 1). As mentioned above, the right half of the brain was analyzed for the cell counting and therefore the numbers of cells obtained were multiplied by 2. The isotropic fractionator technique consists in mechanically dissociating the brain tissue in 40 mM sodium citrate with 1% Triton X-100 using Tenbroeck tissue homogenizers (Herculano-Houzel and Lent, 2005). The homogenization process is done when no more tissue fragments are visible. This process lasted 5–10 mins for the optic tectum, 10–15 min for the cerebellum, and 15–25 min for the telencephalon. By transforming the brain into a suspension of free cell nuclei, the total numbers of cells are estimated by using a fluorescent DNA marker 4′,6-Diamidine-2′-phenylindole dihydrochloride (DAPI). A minimum of four aliquots (10 μl) per brain region are counted using Neubauer improved chamber under a fluorescent Nikon eclipse 80i microscope at 400× magnification (numerical aperture 0.95). The coefficient of variation among the four aliquots were lower than 0.15. To determine the proportion of neurons among our samples, we used immunocytochemical detection of neuronal nuclear antigen NeuN, expressed in the nuclei of most neuronal populations within the brain (Mullen et al., 1992). We note that NeuN is not expressed by Purkinje cells (Mullen et al., 1992) but since this neuronal population does not represent a large fraction of the total cerebellar neurons (Cunha et al., 2020, 2021), not sampling them is not a major issue for our comparative dataset. Our samples were incubated overnight at 10°C in mouse monoclonal antibody anti-NeuN 488 AlexaFluor conjugated (1:300 in phosphate-buffered saline; clone A60, Chemicon; MAB377X) (Olkowicz et al., 2016; Ströckens et al., 2022). At least 500 nuclei were counted to estimate the proportion of neurons in the sample. The isotropic fractionator method has been described by different independent groups (Herculano-Houzel and Lent, 2005; Repetto et al., 2016; Ngwenya et al., 2017; Deniz et al., 2018; Neves et al., 2019) and yield comparable results obtained from stereological methods (Miller et al., 2014; Herculano-Houzel et al., 2015b; Von Bartheld et al., 2016). The number of non-neuronal cells was calculated through subtraction. The densities of neurons and non-neuronal cells were derived by dividing the absolute number of neurons or non-neuronal cells with the mass of the brain regions.

### Statistical analyses

All the statistical analyses were performed in the base package of R 4.1.2 (R Core Team, 2021) with log-transformed data to ensure normalization. The allometric relationships were analyzed by using linear regression models with all individuals for the following comparisons: brain size against body size, neuron number against brain size, neuronal density against brain size, and neuronal density against neuron number. The slopes reported are the scaling exponents obtained by fitting a linear function to log-transformed values. We performed analyses of covariance (ANCOVA) to test for significant differences in the intercepts and slopes of the allometric relationships between males and females.

## Results

The 66 chickens examined in this study varied by 2.23-fold in body mass, 1.48-fold in brain mass, 1.78-fold in the total numbers of neurons of the brain regions examined, and 1.75-fold in the total number of non-neuronal cells of the brain regions examined. Of the brain regions dissected, the cerebellum had the largest range of variation in mass (1.79-fold) while the telencephalon and optic tectum had similar ranges of variation in mass (1.44-fold) (see Table 1). Across our individuals, the numbers of neurons in the brain regions examined had a range of variation between 1.79-fold and 2.46-fold. The numbers of non-neuronal cells in the brain regions analyzed had a range of variation between 2.12 and 3.91 (Table 1).

**TABLE 1:** Average and variation of the mass (g) and numbers of neurons and non-neuronal cells of the regions of interest (ROI) examined.

ROI	Average ± SD	Minimum	Maximum	Variation
**Mass, g**				
Body	1,647 ± 368	1,092	2,430	2.23
Brain	2.859 ± 0.234	2.319	3.431	1.48
Telencephalon	1.558 ± 0.130	1.282	1.847	1.44
Cerebellum	0.390 ± 0.050	0.295	0.529	1.79
Optic tectum	0.333 ± 0.027	0.279	0.403	1.44
Brain remainder	0.579 ± 0.055	0.446	0.741	1.66
**Cells, number**				
Tel, neurons	66,464,632 ± 12,332,337	44,466,113	109,326,190	2.46
Tel, non-neurons	104,414,913 ± 18,249,321	71,112,475	150,622,745	2.12
Cb, neurons	181,813,831 ± 25,236,897	130,517,296	236,161,403	1.81
Cb, non-neurons	23,365,411 ± 6,066,378	9,918,856	38,795,858	3.91
OT, neurons	42,250,728 ± 5,861,537	31,608,284	56,695,922	1.79
OT, non-neurons	25,453,362 ± 4,803,983	15,401,953	38,127,273	2.48

Tel, telencephalon; Cb, cerebellum; OT, optic tectum.

### Brain size correlates with body size

**FIGURE 1:**
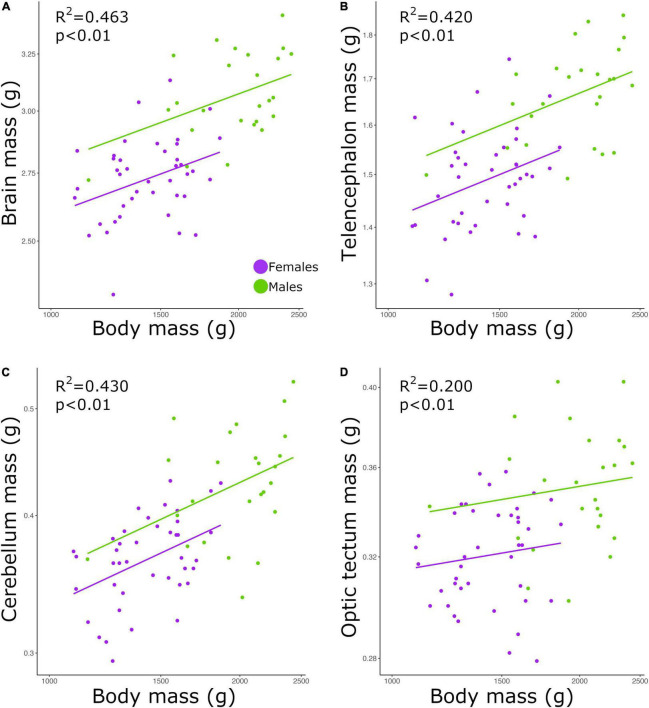
Scatterplots of the masses (g) of the **(A)** brain, **(B)** telencephalon, **(C)** cerebellum, and **(D)** optic tectum against the mass (g) of the body. In all graphs, males are shown in green, and females are shown in purple.

Brain mass and body mass were positively correlated among all individuals (Figure 1A; *p* < 0.01, slope = 0.253; *R*^2^ = 0.463; Table 2). Overall, males had a significantly higher intercept for this relationship when compared with the intercept found for females (ANCOVA, *p* < 0.01; no slope difference; Supplementary Table 2). We found the same statistical pattern for all brain regions dissected. The mass of the telencephalon (Figure 1B; *p* < 0.01, slope = 0.247; *R*^2^ = 0.420; Table 2), cerebellum (Figure 1C; *p* < 0.01, slope = 0.379; *R*^2^ = 0.430; Table 2), and optic tectum (Figure 1D; *p* < 0.01, slope = 0.171; *R*^2^ = 0.200; Table 2) increased as a function of the body mass across all individuals. All brain regions were relatively larger in males than in females (ANCOVA, *p*’s < 0.05; no slope differences; Supplementary Table 2).

**TABLE 2:** Details of the allometric relationships among the measurements examined and including all individuals.

*x*-Axis	*y*-Axis	Intercept	Slope	*F*-stat.	*R* ^2^	*P*-value
Body mass	Brain mass	−0.356	0.253 ± 0.033	57.0	0.463	<0.01
	Tel mass	−0.600	0.247 ± 0.035	48.2	0.420	<0.01
	Cb mass	−1.627	0.379 ± 0.054	50.0	0.430	<0.01
	OT mass	−1.027	0.171 ± 0.041	17.2	0.200	<0.01
Tel mass	Tel #neurons	7.629	0.979 ± 0.244	16.1	0.189	<0.01
	Tel #non-neurons	7.740	1.426 ± 0.188	57.2	0.464	<0.01
	Tel N/g	–	−0.021 ± 0.244	–	–	0.93
	Tel O/g	7.740	0.426 ± 0.188	5.1	0.059	0.03
Cb mass	Cb #neurons	8.556	0.729 ± 0.107	46.5	0.412	<0.01
	Cb #non-neurons	–	0.373 ± 0.275	–	–	0.18
	Cb N/g	8.556	−0.271 ± 0.107	6.4	0.077	0.01
	Cb O/g	7.507	−0.627 ± 0.275	5.2	0.061	0.02
OT mass	OT #neurons	8.060	0.915 ± 0.177	26.8	0.284	<0.01
	OT #non-neurons	7.810	0.859 ± 0.266	10.4	0.126	<0.01
	OT N/g	–	−0.085 ± 0.177	–	–	0.63
	OT O/g	–	−0.141 ± 0.266	–	–	0.60
Tel #neurons	Tel N/g	1.416	0.794 ± 0.051	240.6	0.787	<0.01
Tel #non-neurons	Tel O/g	2.461	0.669 ± 0.044	233.7	0.782	<0.01
Cb#neurons	Cb N/g	5.178	0.423 ± 0.085	24.9	0.269	<0.01
Cb#non-neurons	Cb O/g	0.964	0.925 ± 0.055	280.0	0.811	<0.01
OT#neurons	OT N/g	2.938	0.677 ± 0.062	118.2	0.643	<0.01
OT#non-neurons	OT O/g	1.683	0.837 ± 0.050	275.2	0.808	<0.01

The slopes are the scaling exponents obtained by fitting a linear function to log-transformed values. Tel, telencephalon; Cb, cerebellum; OT, optic tectum; N/g, neuronal density; O/g, non-neuronal density.

### Cell number correlates with brain size

**FIGURE 2:**
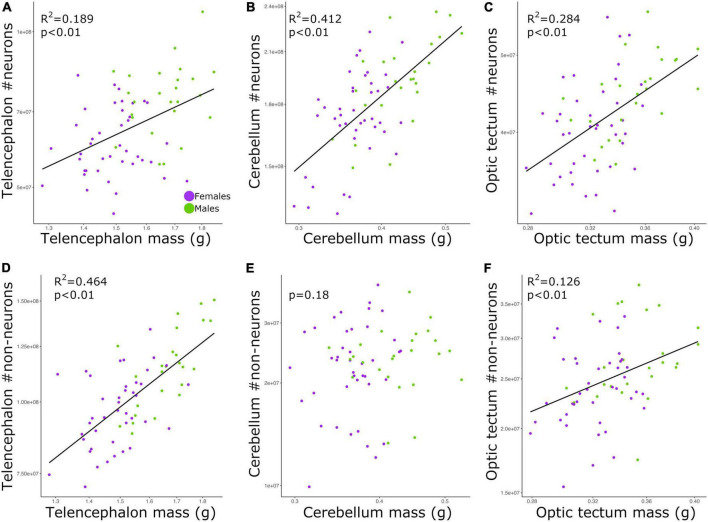
Scatterplots of the **(A)** neuron numbers in the telencephalon against telencephalon mass, **(B)** neuron numbers in the cerebellum against cerebellum mass, **(C)** neuron numbers in the optic tectum against optic tectum mass, **(D)** non-neuronal cell numbers in the telencephalon against telencephalon mass, **(E)** non-neuronal cell numbers in the cerebellum against cerebellum mass, and **(F)** non-neuronal cell numbers in the optic tectum against optic tectum mass. In all graphs, males are shown in green, and females are shown in purple.

The total numbers of neurons in each brain region were positively correlated with the size of all three brain regions (Figures 2A–C; telencephalon: slope = 0.979, *R*^2^ = 0.189; cerebellum: slope = 0.729, *R*^2^ = 0.412; and optic tectum: slope = 0.915, *R*^2^ = 0.284; *p*’s < 0.01; Table 2). For all relationships above, there were no differences in the intercepts and slopes between males and females (ANCOVA, *p*’s > 0.05; Supplementary Table 2). Thus, in contrast to the findings relating to body mass (Figure 1), here, where body mass is not a variable, a single power function describes the relationship between brain region mass and number of neurons across males and females.

For the relative numbers of non-neuronal cells, we found significant relationships for the telencephalon and optic tectum (Figures 2D,F; *p*’s < 0.01) but not the cerebellum (Figure 2E, *p* = 0.18; Table 2). The numbers of non-neuronal cells in the telencephalon and optic tectum increased as a function of the size of the respective brain region among all individuals (telencephalon: slope = 1.426, *R*^2^ = 0.464; optic tectum: slope = 0.859, *R*^2^ = 0.126; Table 2). For both brain regions, the intercepts and slopes were not significantly different between males and females (ANCOVA, *p*’s > 0.05; Supplementary Table 2).

### The relationship between cell density and brain mass is weak and varies between brain regions

**FIGURE 3:**
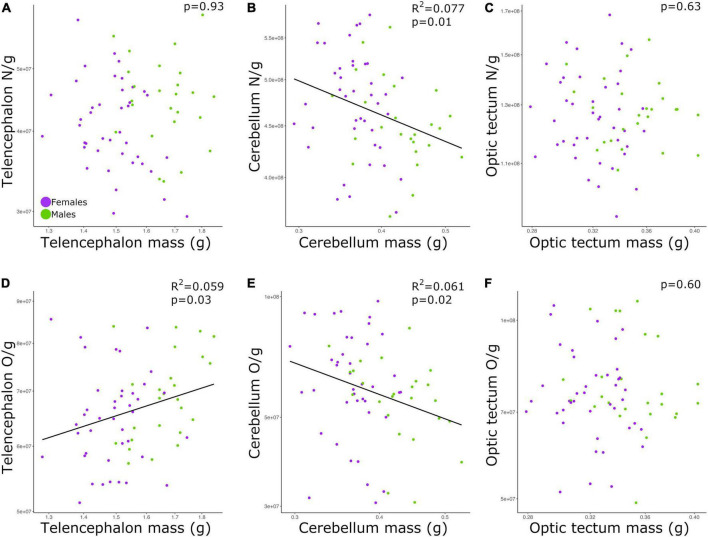
Scatterplots of **(A)** neuronal density in the telencephalon against telencephalon mass, **(B)** neuronal density in the cerebellum against cerebellum mass, **(C)** neuronal density in the optic tectum against optic tectum mass, **(D)** non-neuronal density in the telencephalon against telencephalon mass, **(E)** non-neuronal density in the cerebellum against cerebellum mass, **(F)** non-neuronal density in the optic tectum against optic tectum mass. In all graphs, males are shown in green, and females are shown in purple. N/g, neuronal density; O/g, non-neuronal density.

The neuronal densities in the telencephalon and optic tectum were not significantly correlated with the mass of the corresponding brain region among all individuals (Figures 3A,C; telencephalon: *p* = 0.93; optic tectum: *p* = 0.63; Table 2). Conversely, the neuronal density in the cerebellum was negatively correlated with the mass of the cerebellum (Figure 3B; *p* = 0.01, slope = −0.271; *R*^2^ = 0.077; Table 2) and no significant differences were detected in the intercept and slope between sexes (ANCOVA, *p* > 0.05; Supplementary Table 2).

While the density of non-neuronal cells in the telencephalon increased as a function of the telencephalon mass (Figure 3D; *p* = 0.03, slope = 0.426; *R*^2^ = 0.059; Table 2), the density of non-neuronal cells in the cerebellum decreased as function of the cerebellum mass among all individuals (Figure 3E; *p* = 0.02, slope = −0.627; *R*^2^ = 0.061; Table 2). For both relationships, the intercepts and slopes were not significantly different between sexes (ANCOVA, *p*’s > 0.05; Supplementary Table 2). For the optic tectum, the relationship between the density of non-neuronal cells and the mass of the brain region did not reach significance (Figure 3F; *p* = 0.60; Table 2).

### Cell density correlates with absolute cell number

**FIGURE 4:**
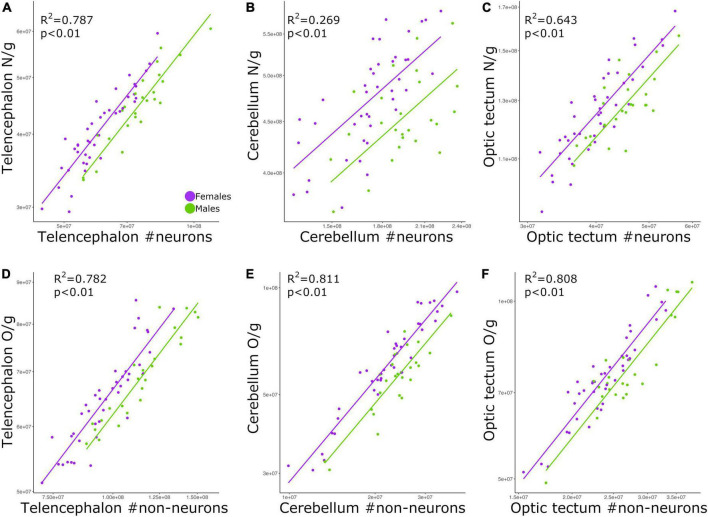
Scatterplots of **(A)** neuronal density in the telencephalon against neuron numbers in the telencephalon, **(B)** neuronal density in the cerebellum against neuron numbers in the cerebellum, **(C)** neuronal density in the optic tectum against neuron numbers in the optic tectum, **(D)** non-neuronal density in the telencephalon against non-neuronal cell numbers in the telencephalon, **(E)** non-neuronal density in the cerebellum against non-neuronal cell numbers in the cerebellum, and **(F)** non-neuronal density in the optic tectum against non-neuronal cell numbers in the optic tectum. In all graphs, males are shown in green, and females are shown in purple. N/g, neuronal density; O/g, non-neuronal density.

The relationship between neuronal density and absolute number of neurons was significant for all brain regions examined: telencephalon (Figure 4A; *p* < 0.01, slope = 0.794; *R*^2^ = 0.787; Table 2), cerebellum (Figure 4B; *p* < 0.01, slope = 0.423; *R*^2^ = 0.269; Table 2), and optic tectum (Figure 4C; *p* < 0.01, slope = 0.677; *R*^2^ = 0.643; Table 2). For the three brain regions, males had relatively lower neuronal densities than females (ANCOVA, *p*’s < 0.05; no slope differences; Supplementary Table 2).

Just as we found for neuronal density, the relationship between non-neuronal density and absolute number of non-neuronal cells was significant for the telencephalon (Figure 4D; *p* < 0.01, slope = 0.669; *R*^2^ = 0.782; Table 2), cerebellum (Figure 4E; *p* < 0.01, slope = 0.925; *R*^2^ = 0.811; Table 2), and optic tectum (Figure 4F; *p* < 0.01, slope = 0.837; *R*^2^ = 0.808; Table 2). For all three brain regions, males had relatively lower non-neuronal densities than females (ANCOVA, *p*’s < 0.05; no slope differences; Supplementary Table 2).

## Discussion

Here we examined the allometric relationships of the brain within a population of 66 highly variable wild-domestic advanced intercross chickens. The coefficients of variation in brain mass and total numbers of neurons and non-neuronal cells in our intercross population were on average 2.9× higher than what was observed within a population of laboratory mice (Herculano-Houzel et al., 2015a). We show that the whole brain and the three brain regions examined (telencephalon, cerebellum, and optic tectum) significantly increase in size with body size. In a similar fashion, the numbers of neurons and non-neuronal cells generally increased as a function of the brain mass but with relatively low levels of coefficients of determination. Our data reveal that neuronal density is strongly and positively correlated with the absolute number of neurons among chickens, which is to say that within our population, more neurons in the brain reflect higher neuronal densities. Our dataset reveals some differences in the allometric scaling of the brain between males and females and indicates that including both sexes into the sample can be critical to obtaining a complete picture of the allometric scaling of the brain within a population.

One caveat to mention before discussing the results is that our intercross has a very clear population structure in that all individuals are in essence full siblings with one another. Although this controlled relatedness structure allows us to determine how the brain anatomy changes between individuals in this study, we acknowledge that this is not entirely representative of the relatedness seen in wild populations. Nevertheless, our populational structure should not affect our conclusions in terms of how body size, brain size, and cellular composition vary with one another, but it will be interesting to juxtapose the results found here with those from a highly variable population in natural conditions.

### Brain size increases with body size

In this study, we found a positive correlation between body mass and brain mass. Our results also show that male chickens had relatively larger brains than female chickens. However, as show in Figure 1, this difference appears to be simply caused by the fact that males have larger bodies, especially given that the slopes for both sexes are similar. To our knowledge, there is no agreement on whether brain size significantly covaries with body size within a population (Gonda et al., 2009; Kolm et al., 2009; Herculano-Houzel et al., 2015a; Kverková et al., 2020). While it has been reported that larger individuals of pumpkinseed sunfish (*Lepomis gibbosus*) (Axelrod et al., 2018), brown trout (*Salmo trutta*) (Kolm et al., 2009), and geckoes (*P. picta*) (Kverková et al., 2020) have overall larger brains, among individuals of mice (*M. musculus*) the size of the brain was not significantly explained by the size of the body (Herculano-Houzel et al., 2015a). Apart from differences intrinsic to each species, the divergent result on mice might be due to two reasons. First, the study on mice neither includes wild caught specimens nor different breeding populations, which likely diminishes the diversity in traits (e.g., brain size) within the population analyzed. Second, only male mice were examined in the study by Herculano-Houzel et al. (2015a). Given the differences we found in the relative brain mass between males and females, the lack of one sex in the analysis of Herculano-Houzel et al. (2015a) could have potentially created a bias in their result. Although in our study we analyzed a single captive population (F18), our individuals were originated from two phenotypically different lines, meaning that within our one population the diversity of traits is maximized. Thereby, our results reveal that within a population that has high variation in body size and brain size, larger individuals tend to have larger brains. This finding, however, does not necessarily mean that brain size is entirely dependent on body size or vice-versa. We have previously found separate, independent genetic architectures for brain mass and body mass in chickens, indicating that selection on brain mass is not limited by body mass, when considering inter-population variation (Henriksen et al., 2016). Despite the fact that body size explained 46% of the variation in brain mass—a relatively high value considering that variation within species likely includes a large amount of non-genetic variation, our results still reveal that both traits are not strictly dependent on one another.

### Larger brains have more neurons

Among our individuals, the numbers of neurons were significantly and positively correlated with the sizes of the brain regions examined (Figure 2). However, for all relationships there was high scatter around the allometric lines. The same pattern was seen for non-neuronal cells in the telencephalon and optic tectum, where non-neuronal cell number increased with brain size. However, for the cerebellum there was no correlation between the size of this brain region and the number of non-neuronal cells. The finding that brain size explained on average ∼29% of the variation in cell numbers suggests that both traits are not completely tied to one another. This is supported by genetic studies in mice where it was shown that independent loci modulate the volume and neuron number of the striatum, demonstrating that selection can act independently on brain region size and neuron number (Rosen and Williams, 2001). Given the evidence that brain size and cell number have at least some degree of independence within species, our chicken intercross model could be an excellent model to quantify the genetic overlap between brain size and cell number across brain regions. Additionally, other parameters within the brain might also contribute to the changes in brain size. Neuron size, for instance, has been suggested to reflect changes in the size of the brain (Smith et al., 1997; Freas et al., 2013; Chang et al., 2020). It has been recently shown that galliform species with larger cerebellum have lower neuronal densities and larger neurons (Cunha et al., 2020; Racicot et al., 2021; Kverková et al., 2022). Due to the lack of quantitative, neuroanatomical data within species, at this point it is difficult to determine what parameter within the brain, rather than neuron number, is highly correlated with brain size within a population. An empirical test of whether a broad range of brain parameters, including neuronal size, change in concert with brain size within a population would provide insights into the allometric relationships of the brain.

We also note that for the telencephalon, the number of non-neuronal cells increased at a faster rate relative to the increase observed in the mass of the telencephalon. Conversely, the number of neurons in the telencephalon increased isometrically with the size of the telencephalon. This means that larger telencephalon contains disproportionate numbers of non-neuronal cells when compared to individuals with smaller telencephalon. For the optic tectum, however, both neurons and non-neuronal cells increased in number at similar rates relative to the increase observed in the size of the optic tectum. The finding that relative numbers of neurons and non-neuronal cells increase at different rates depending on the brain region analyzed aligns with what has been found across different species (see review Herculano-Houzel, 2014). Therefore, even within species, the proportion between the numbers of neurons and non-neuronal cells in different brain regions is not explained by a single function relative to brain size. In this study, we did not discriminate the different types of non-neuronal cells within the brain. For instance, the steep increase in the numbers of non-neuronal cells relative to the size of the telencephalon could be mostly due to an increase in the numbers of oligodendrocytes and thus more myelin per axon. That being said, until we have detailed quantitative data on the numbers of different types of non-neuronal cells in chickens, we cannot draw any further conclusions on why individuals with larger telencephalon have a disproportionate increase in the number of non-neuronal cells.

### More cells mean higher cell density

By far the strongest relationships found in this study was when correlating the densities of neurons or non-neuronal cells with the absolute numbers of neurons and non-neuronal cells, respectively (Figure 4). Therefore, chickens with more neurons in the brain have relatively higher neuronal densities than chickens with less neurons, a similar pattern to what has been reported in mice (Herculano-Houzel et al., 2015a). This allometric pattern contrasts with what has been found across species, where neuronal density is negatively correlated with brain size and the absolute number of neurons (Herculano-Houzel et al., 2006, 2014; Sarko et al., 2009; Neves et al., 2014; Olkowicz et al., 2016). Here we find that among chickens, neuronal density is weakly correlated with brain size (Figure 3). Indeed, for the six relationships in which neuronal density is correlated with brain size (Figure 3), only three reached levels of significance lower than 0.05, with two of them having values higher or equal than 0.02. Thus, our results suggest that across our individuals cell densities and brain region mass are not linked to one another. On the other hand, the absolute number of neurons was strongly and positively correlated with neuronal density (Figure 4). We note that relative neuronal density in the cerebellum varied with lower coefficient of determination than what was observed in other brain regions or with non-neuronal cells (Figure 4), demonstrating that neuronal and non-neuronal scaling rules vary between brain regions. Regardless of the differences between brain regions, our finding suggests that different mechanisms to change neuronal density in the brain, relative to the absolute number of neurons, exist between populations/within-species vs. across species.

Neuronal density has been extensively used as a brain parameter to indirectly estimate the average size of neurons (Bandeira et al., 2009; Herculano-Houzel, 2011b; Mota and Herculano-Houzel, 2014). More specifically, brains with high neuronal densities are thought to have small neurons (Herculano-Houzel et al., 2014; Mota and Herculano-Houzel, 2014). As a result, we can infer that chickens with more neurons in the brain have relatively high neuronal densities and therefore small neurons when compared to chickens with less neurons in the brain. Moreover, because female chickens had relatively higher neuronal densities than males, we could also expect to find smaller neurons in female chickens than male chickens. However, we urge caution with this conclusion given that recent quantitative studies demonstrated that different neuronal types within the brain can vary in number and size at different rates (Cunha et al., 2020, 2021). Although it is not entirely clear how neuronal cell size affects brain prowess, the inverse relationship between neuronal density and average size of neurons among individuals from the same population could be due to size and metabolic limitations. There is evidence that neuron size correlates positively with energy consumption (Howarth et al., 2012; Niven, 2016), suggesting that having smaller neurons in the brain might be more energy efficient than having larger neurons (Niven and Farris, 2012; Sengupta et al., 2013; von Eugen et al., 2022).

As mentioned above, the individuals in this study are from an intercross between red junglefowl (wild) and White Leghorn (domestic) chickens. It has been shown that domestication in the chicken appears to specifically affect the anatomy of the cerebellum (Henriksen et al., 2016; Racicot et al., 2021). White Leghorns have a larger cerebellum with proportionally more neurons and relatively larger granule cells when compared with red junglefowl (Henriksen et al., 2016; Racicot et al., 2021). Accordingly, the relatively high scatter around the allometric line for the relationship between neuronal density and neuron numbers in the cerebellum (Figure 4B) could be the result of the effects of domestication on the average size of granule cells among some of our individuals. It is worth noting that we did not find the same scatter pattern for non-neuronal cells (Figure 4E), possibly meaning that non-neuronal cells in the cerebellum are not affected by the process of domestication in the chicken.

## Conclusion

Overall, we conclude that within a population of highly variable chickens, larger individuals tend to have larger brains and more neurons. Among chickens, neuronal density was strongly and positively associated with neuron number, an opposite scaling pattern to that observed in interspecific analyzes (Herculano-Houzel et al., 2014; Olkowicz et al., 2016). The extent to which neuronal density reflects changes in other brain parameters is still unknown but would provide great insights into the brain evolution within population.

## Data availability statement

The original contributions presented in this study are included in the article/Supplementary material, further inquiries can be directed to the corresponding authors.

## Ethics statement

This animal study was reviewed and approved by the local Ethical Committee of the Swedish National Board for Laboratory Animals.

## Author contributions

DW and RH: funding acquisition. FC, DS-H, RC, DW, and RH: data collection. FC: data analysis and writing—initial draft. FC, DS-H, DW, and RH: conceptualization and writing—final draft. All authors had full access to the data in the study, take responsibility for the integrity of the data, accuracy of the data analysis, revised the initial and final draft, and gave final approval for publication.
