# Supplementary material for: Neuronal and non-neuronal scaling across brain regions within an intercross of domestic and wild chickens
Source: Front Neuroanat. 2022 Nov 25;16:1048261. doi: 10.3389/fnana.2022.1048261 (PMC9732670; doi:10.3389/fnana.2022.1048261)
Supplement: Supplementary file 2 [file Table_2.docx]

**Table S2.** P-values from the analyses of covariance (ANCOVA) testing for differences in the intercept (int) and slope between males and females for the relationships performed. Tel=telencephalon; Cb=cerebellum; OT=optic tectum; N/g=neuronal density; O/g=non-neuronal density.

|  |  | **ANCOVA, p-value** | |
| --- | --- | --- | --- |
| **x-axis** | **y-axis** | int | slope |
| **Body mass** | Brain mass | **<0.01** | 0.63 |
|  | Tel mass | **<0.01** | 0.79 |
|  | Cb mass | **0.03** | 0.72 |
|  | OT mass | **<0.01** | 0.86 |
| **Tel mass** | Tel #neurons | 0.78 | 0.41 |
|  | Tel #non-neurons | 0.60 | 0.10 |
|  | Tel N/g | - | - |
|  | Tel O/g | 0.60 | 0.10 |
| **Cb mass** | Cb #neurons | 0.89 | 0.99 |
|  | Cb #non-neurons | - | - |
|  | Cb N/g | 0.88 | 0.99 |
|  | Cb O/g | 0.89 | 0.62 |
| **OT mass** | OT #neurons | 0.41 | 0.91 |
|  | OT #non-neurons | 0.13 | 0.94 |
|  | OT N/g | - | - |
|  | OT O/g | - | - |
| **Tel #neurons** | Tel N/g | **<0.01** | 0.49 |
| **Tel #non-neurons** | Tel O/g | **<0.01** | 0.34 |
| **Cb #neurons** | Cb N/g | **<0.01** | 0.11 |
| **Cb #non-neurons** | Cb O/g | **<0.01** | 0.99 |
| **OT #neurons** | OT N/g | **<0.01** | 0.12 |
| **OT #non-neurons** | OT O/g | **<0.01** | 0.89 |

**Supplementary Figure**. **A.** Dorsal view of the whole brain of a chicken and **B.** the four brain regions dissected. 1=telencephalon; 2=optic tectum; 3=cerebellum; 4=brain remainder.
