# Supplementary figures and images for: Neuronal and non-neuronal scaling across brain regions within an intercross of domestic and wild chickens
Source: Front Neuroanat. 2022 Nov 25;16:1048261. doi: 10.3389/fnana.2022.1048261 (PMC9732670; doi:10.3389/fnana.2022.1048261)

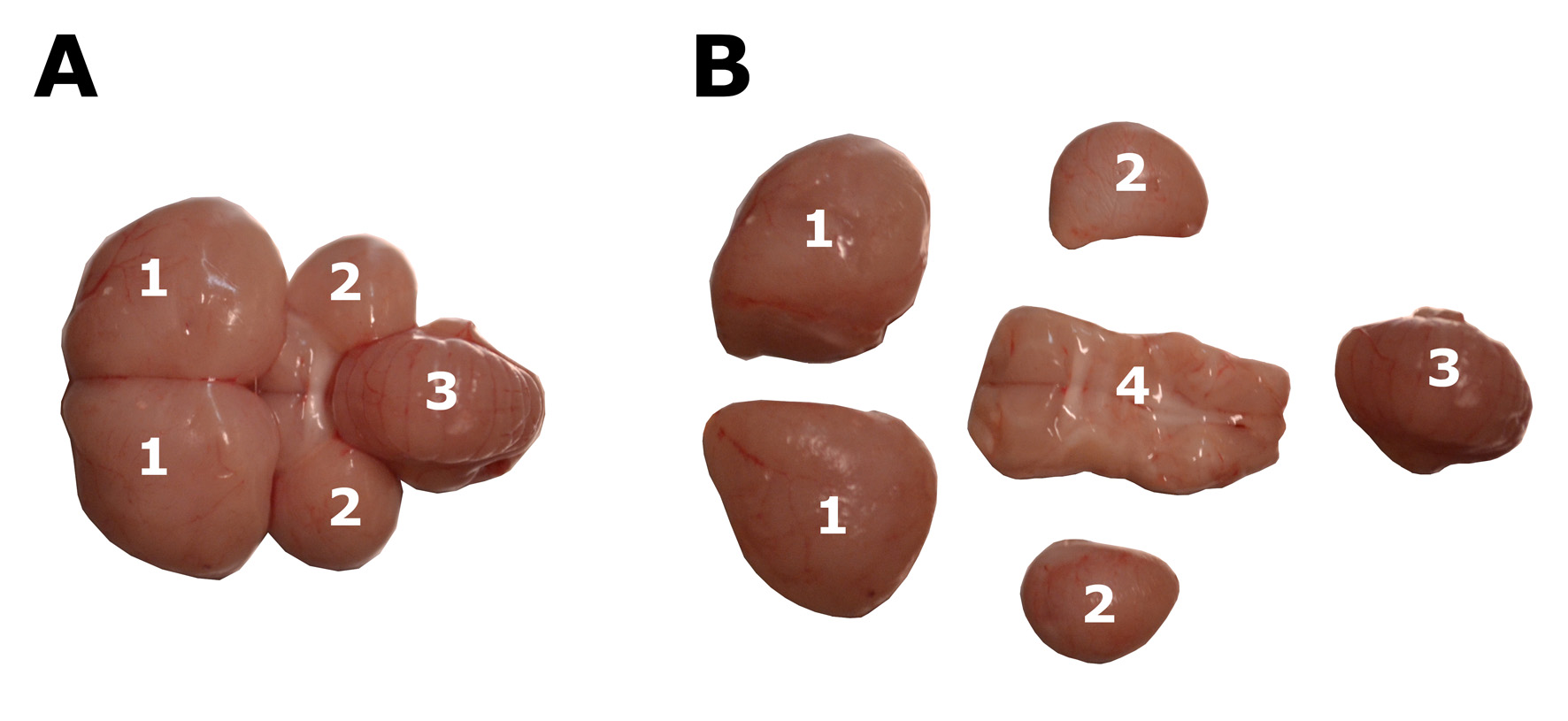

Supplement: Supplementary file 3 [file Image_1.JPEG]
